# The mediating role of inflammation in the association between pregnancy loss history and gestational diabetes mellitus

**DOI:** 10.1186/s13098-023-01106-w

**Published:** 2023-06-21

**Authors:** Qiong Li, Haixia Wang, Lijun Sun, Peng Wang, Wanjun Yin, Shuangshuang Ma, Ruixue Tao, Jinfang Ge, Peng Zhu

**Affiliations:** 1 Department of Maternal, Child and Adolescent Health, School of Public Health, Anhui Medical University, Hefei, China; 2 MOE Key Laboratory of Population Health Across Life Cycle, Hefei, China; 3 NHC Key Laboratory of Study on Abnormal Gametes and Reproductive Tract, Anhui Medical University, Hefei, China; 4 Anhui Provincial Key Laboratory of Population Health and Aristogenics, Anhui Medical University, Hefei, China; 5 Department of Gynecology and Obstetrics, Hefei First People’s Hospital, Hefei, China; 6 School of Pharmacy, Anhui Medical University, Hefei, China

**Keywords:** Pregnancy loss, Induced abortion, Miscarriage, Gestational diabetes mellitus, Inflammation

## Abstract

**Background:**

To assess the association of pregnancy loss history with an elevated risk of Gestational diabetes mellitus (GDM) and to investigate whether this association was mediated by high-sensitivity C-reactive protein (hs-CRP).

**Methods:**

We prospectively collected venous blood and pregnancy loss history information from 4873 pregnant women at 16–23 weeks of gestation from March 2018 to April 2022. Hs-CRP concentrations were measured from collected blood samples. A 75 g fasting glucose test was performed at 24 to 28 weeks of gestation for the diagnosis of GDM, with data obtained from medical records. Multivariate linear or logistic regression models and mediation analysis were used to examine the relationships between pregnancy loss history, hs-CRP, and GDM.

**Results:**

A multivariable-adjusted logistic regression analysis revealed that compared with pregnant women with no induced abortion history, subjects with 1 and ≥ 2 induced abortions had a higher risk for GDM (RR = 1.47, 95% CI = 1.19–1.81; RR = 1.63, 95% CI = 1.28–2.09). Additionally, the mediation analysis indicated this association was mediated by an increased hs-CRP level with a 20.4% of indirect effect ratio. However, no significant association between a history of miscarriage and the prevalence of GDM was observed.

**Conclusions:**

A history of induced abortion was significantly associated with an increased risk of GDM, and this association occurred in a dose-response effect. Hs-CRP may be accounted for a mediation effect in the pathways linking induced abortion history with GDM.

## Introduction

Gestational diabetes mellitus (GDM), defined as glucose intolerance with first onset or recognition during pregnancy, has emerged as the most common metabolic complication, affecting 7–25% of pregnancies worldwide [1–3]. More seriously, the incidence of GDM continues to increase, which has persistent effects on long-term health in both mothers and their offspring. There is increasing evidence that maternal GDM has been associated with adverse outcomes, including cardiovascular disease, type 2 diabetes mellitus [4], and offspring’s risk of cardiometabolic, growth, and neurodevelopment [5–7]. Given the burden of GDM, further efforts are needed to improve GDM prevention.

Pregnancy loss, both naturally occurring and induced, is common in obstetrics and gynecology. An estimated 23 million miscarriages occur every year worldwide [8]. Several studies have shown that miscarriage, especially recurrent miscarriage, is associated with the risk of later cardiovascular disease and diabetes [9–11]. Induced abortion (hereafter abortion) is one of the most common medical interventions widely used to terminate unwanted pregnancies [12]. Some previous studies reported that women with a history of abortion are at higher risk of developing metabolic diseases [13]–[14]. However, epidemiological studies which focused on the impact of pregnancy loss history on maternal risk of GDM in subsequent pregnancies were still limited.

The inflammatory response may be one of the potential mechanisms for the association between pregnancy loss history and GDM. In miscarriage, increased regulation and function of dNK cells can induce an anti-inflammatory state at the maternal-fetal interface [15]. In abortion, nonstandard procedures may cause infection by leaving dead tissue in the uterus [16]. Inflammation may cause glucose metabolism disorder during pregnancy. One systematic review found that high-sensitivity C-reactive protein (hs-CRP) can be considered a potential inflammatory marker in predicting GDM [17]. Hence, a better understanding of whether inflammation in the context of pregnancy loss history affected the risk of GDM, which would facilitate the development of more targeted intervention and prevention strategies.

The objectives of this study were (1) to explore the association between pregnancy loss history and GDM; (2) to assess whether such association was mediated by hs-CRP.

## Methods

### Participants

From March 2018 to April 2022, the participants are derived from the prospective cohort study in 3 centers including Anhui Women and Child Health Care Hospital, The First People’s Hospital of Hefei City, and The First Affiliated Hospital of Anhui Medical University. Women aged ≥ 18 years, 16–23 gestation weeks, no communication problems, residents of Hefei, and singleton pregnancy were recruited. A standardized questionnaire was completed and blood samples were collected at enrollment. A fasting glucose tolerance test with 75 g glucose was performed at 24–28 weeks for the diagnosis of GDM, and data were obtained from the medical electronic system. Finally, 4873 pregnant women were included in the analysis. Written informed consent was obtained from all pregnant women and the study was approved by the Ethics Committee of Anhui Medical University (number: 20,180,092).

### Data on pregnancy loss history

At enrollment, the history of previous pregnancy loss history (yes or no) and the number of miscarriages and abortions (0,1,≥2) were face-to-face interviewed. Their reproductive histories prior to this pregnancy were determined by maternal report and validated by medical records.

### Assessment of GDM

Pregnant women completed a 75-g oral-glucose-tolerance test (OGTT) in 24–28 gestation weeks and the diagnosis of GDM was based on the criteria proposed by the International Association of Diabetes in Pregnancy Study Groups (IADPSG): fasting plasma glucose (FPG) ≥ 5.1 mmol/L, and/or 1 h plasma glucose (1-h PG) ≥ 10.0 mmol/L, and/or 2 h plasma glucose (2-h PG) ≥ 8.5 mmol/L [18].

### The measurement of maternal hs-CRP concentration

Blood samples were collected at enrollment(16–23 gestation weeks). The maternal hs-CRP was measured using the enzyme-linked immunosorbent assay (ELISA) kits (Cusabio Biotech, Wuhan, China) according to the manufacturer’s instructions. The minimum detectable concentration was 0.02 µg/ml, and the inter-class and intra-class coefficients of variation were less than 10%.

### Confounding variables

All potential covariates were selected a priori. Potential confounders in this study included maternal age (years), education level (< 12, ≥ 12 years), pre-pregnancy BMI (kg/m^2^), parity (nulliparous or multiparous), family history of diabetes (yes or no).

### Statistical analysis

Data of baseline characteristics are presented as the percentage. Multiple logistic regression analysis was used to estimate relative risk (RR) and 95% confidence interval (CI) for the association between pregnancy loss history and GDM. Multivariable-adjusted model included maternal age, BMI, educational level, parity, and family history of diabetes. Multiple linear regression models were used to analyze the associations of change in hs-CRP concentrations with the number of miscarriages and abortions. We also performed logistic regression models to explore the association of hs-CRP concentrations with GDM. All regression models were adjusted for the aforementioned confounders. Mediation analysis was performed using the SPSS PROCESS plug-in to evaluate the role of hs-CRP in the association between pregnancy loss history and GDM. All data analyses were conducted in SPSS version 26.0 software (IBM Corp, Armonk, NY, USA).

## Results

### Baseline characteristics of the study population

Table 1 summarized the general characteristics of the study populations according to pregnancy loss history. Of the 4873 pregnant women included, 2129(43.7%) had a pregnancy loss history. There were 1280(26.3%) of one abortion and 625(12.8%) of more than two abortions. 414(8.5%) with one miscarriage and 117(2.4%) with more than two miscarriages. Compared to women without pregnancy loss history, those with pregnancy loss history were more likely to be older, to be multiparous, and to have obesity. The levels of FPG, 1-h PG, 2-h PG, and incidence of GDM in women with pregnancy loss history were higher than those without pregnancy loss history.

**Table 1:** Characteristics of the Participants According to History of Pregnancy Loss (N = 4873)

	Pregnancy loss			Induced abortion				Miscarriage		
Characteristics	No (n = 2744)	Yes (n = 2129)		0 (n = 2968)	1 (n = 1280)	≥ 2 (n = 625)		0 (n = 4342)	1 (n = 414)	≥ 2 (n = 117)
Age, y										
< 35	2555(93.1)	1691(79.4)		2751(92.7)	1077(84.1)	418(66.9)		3808(87.7)	350(84.5)	88(75.2)
≥ 35	189(6.9)	438(20.6)		217(7.3)	203(15.9)	307(33.1)		534(12.3)	64(15.5)	29(24.8)
Education level, y										
< 12	1940(70.7)	1698(79.8)		2104(70.9)	1011(79.0)	523(83.7)		3226(74.3)	324(78.3)	88(75.2)
≥ 12	804(29.3)	431(20.2)		864(29.1)	269(21.0)	102(16.3)		1116(25.7)	90(21.7)	29(24.8)
Pre-pregnancy BMI, kg/m^2^										
< 18.5	408(14.9)	230(10.8)		428(14.4)	155(12.1)	55(8.8)		585(13.5)	44(10.6)	9(7.7)
18.5–24.9	2063(75.2)	1595(74.9)		2235(75.3)	950(74.2)	473(75.7)		3262(75.1)	307(74.2)	89(76.1)
≥ 25	273(9.9)	304(14.3)		305(10.3)	175(13.7)	97(15.5)		495(11.4)	63(15.2)	19(16.2)
Parity										
Nulliparous	1759(64.1)	33(1.6)		1772(59.7)	9(0.7)	11(1.8)		1774(40.9)	11(2.7)	7(6.0)
Multiparous	985(35.9)	2096(98.4)		1196(40.3)	1271(99.3)	614(98.2)		2568(59.1)	403(97.3)	110(94.0)
Family history of diabetes										
Yes	225(8.2)	243(11.4)		245(8.3)	136(10.6)	87(13.9)		402(9.3)	51(12.3)	15(12.8)
No	2519(91.8)	1886(88.6)		2723(91.7)	1144(89.4)	538(86.1)		3940(90.7)	363(87.7)	102(87.2)
FPG, mmol/L	4.52 ± 0.45	4.58 ± 0.46		4.52 ± 0.44	4.58 ± 0.45	4.61 ± 0.48		4.54 ± 0.45	4.58 ± 0.45	4.58 ± 0.42
1 h-PG, mmol/L	7.22 ± 1.67	7.59 ± 1.74		7.32 ± 1.62	7.54 ± 1.73	7.82 ± 1.81		7.42 ± 1.68	7.63 ± 1.77	7.61 ± 1.57
2 h-PG, mmol/L	6.32 ± 1.22	6.60 ± 1.31		6.38 ± 1.21	6.55 ± 1.30	6.69 ± 1.35		6.44 ± 1.25	6.65 ± 1.31	6.68 ± 1.19
GDM										
Yes	404(14.7)	437(20.5)		431(14.5)	260(20.3)	150(24.0)		730(16.8)	89(21.5)	22(18.8)
No	2340(85.3)	1692(79.5)		2537(85.5)	1020(79.7)	475(76.0)		3612(83.2)	325(78.5)	95(81.2)

*BMI* body mass index, *FPG* fasting plasma glucose, *1 h-PG* 1 h plasma glucose, *2 h-PG* 2 h plasma glucose, *GDM* gestational diabetes mellitus

### Association of pregnancy loss history with GDM

Table 2 shows that the associations between pregnancy loss history and GDM. There was a significant association between pregnancy loss history and the prevalence of GDM in logistic regression models (crude model: RR = 1.50, 95% CI = 1.29–1.74; adjusted model: RR = 1.37, 95% CI = 1.12–1.67). However, the association was different for abortion and miscarriage. We observed that in the adjusted model, compared with pregnant women with no abortion history, subjects with 1 and ≥ 2 abortions yielded 47–63% higher risk for GDM (RR = 1.47, 95% CI = 1.19–1.81; RR = 1.63, 95% CI = 1.28–2.09). However, after full adjustment, no associations were found for miscarriage.

**Table 2:** Risk of prevalent GDM according to the history of pregnancy loss

Group		n(%)		Crude model		Adjusted model
Pregnancy loss						
No		417(15.2)		1.00 (ref.)		1.00 (ref.)
Yes		446(20.9)		1.50(1.29, 1.74)		1.37(1.12, 1.67)
*P*				< 0.001		0.002
Induced abortion^*^						
0		444(15.0)		1.00 (ref.)		1.00 (ref.)
1		263(20.5)		1.50(1.27, 1.78)		1.47(1.19, 1.81)
≥ 2		156(24.9)		1.86(1.51, 2.29)		1.63(1.28, 2.09)
*P*_trend_				< 0.001		< 0.001
Miscarriage^†^						
0		750(17.3)		1.00 (ref.)		1.00 (ref.)
1		90(21.7)		1.36(1.06, 1.74)		1.25(0.96, 1.61)
≥ 2		23(19.7)		1.15(0.72, 1.83)		0.87(0.53, 1.40)
*P*_trend_				0.165		0.240

Adjusted model: Adjusted for maternal age, pre-pregnancy BMI, educational level, parity, and family history of diabetes

^*^Plus adjustment for history of miscarriage

^†^Plus adjustment for history of induced abortion

### Associations of hs-CRP with the number during abortions or miscarriages

**Fig. 1:**
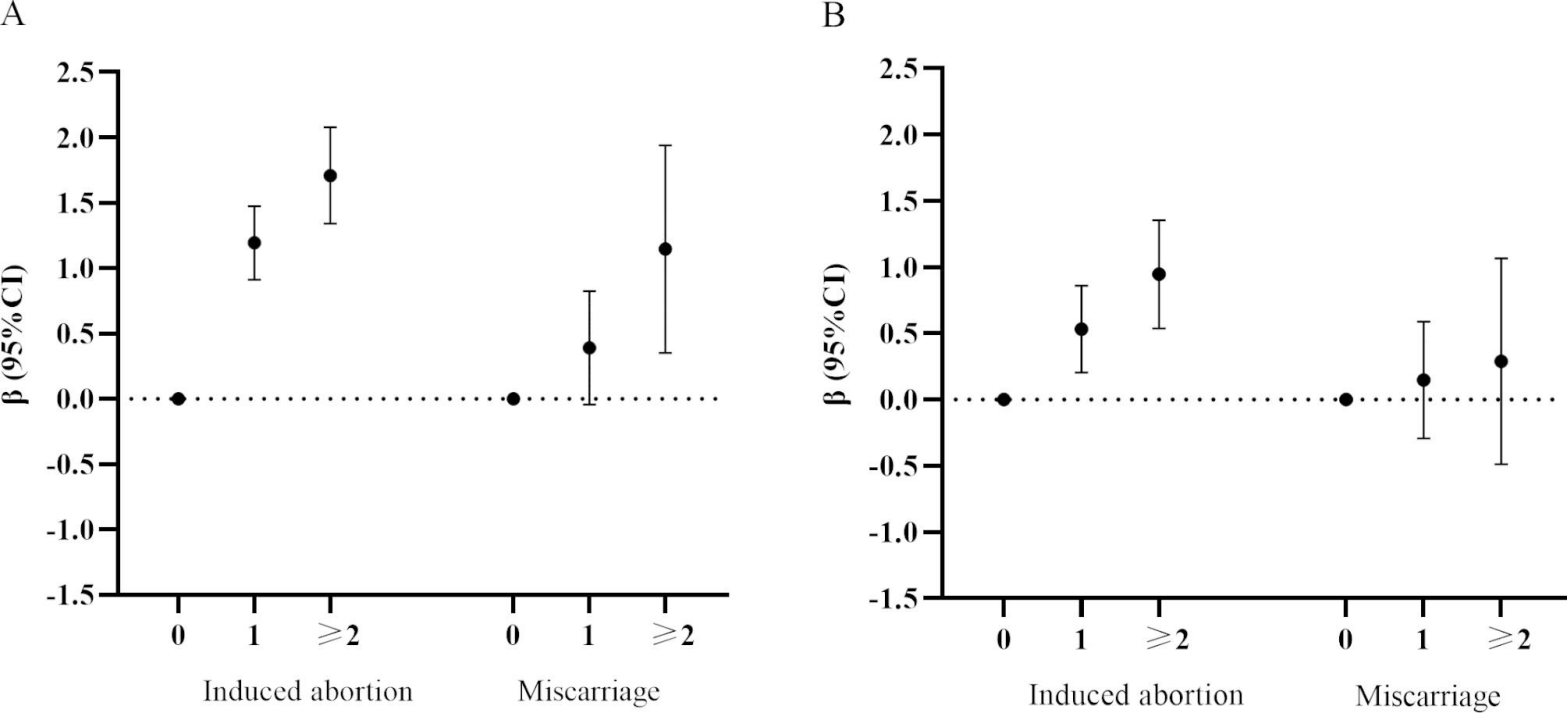
The associations of change in hs-CRP concentrations with the number during induced abortions or miscarriages Graphs show the estimated change in hs-CRP concentrations (β) and 95%CI for the crude model **(A)** and adjusted model **(B)**. Models were adjusted for maternal age, pre-pregnancy BMI, educational level, parity, and family history of diabetes

As shown in Fig. 1, a positive association was found between the number of abortions and the change in hs-CRP concentrations. After adjustments were made for confounders, there was a significant increase in hs-CRP concentrations across increasing abortion history numbers (Fig. 1B). In the adjusted model, compared with pregnant women with no abortion history, subjects with 1 and ≥ 2 abortions yielded higher concentration change for hs-CRP (β = 0.53, 95% CI = 0.2–0.86; β = 0.95, 95% CI = 0.54–1.35). However, after full adjustment, no associations were found for miscarriage.

### Association between hs-CRP concentration and risk of GDM

The associations between hs-CRP and GDM risks are presented in Table 3. The prevalence of GDM showed an upward trend when hs-CRP concentration gradually increased. We observed that significant increase in the risk of GDM across tertiles and quartiles of hs-CRP concentration, with a dose-response trend in both crude and adjusted models (all P for trend < 0.01). In the continuous analyses, each 1-unit in log-transformed values of hs-CRP was associated with a 12% (95%CI: 1.10, 1.14) increase in the risk of GDM with adjustment for covariates.

**Table 3:** Association between hs-CRP concentration and risk of GDM

hs-CRP (mg/L)	n(%)	RR(95% CI)		
		Crude model		Adjusted model
Log-transformed hs-CRP	841(17.3)	1.13(1.11, 1.15)		1.12(1.10, 1.14)
T1(< 2.0)	169(10.4)	1.00 (ref.)		1.00 (ref.)
T2(2.0-4.3)	248(15.2)	1.54(1.25, 1.90)		1.41(1.14, 1.74)
T3(≥ 4.3)	424(26.3)	3.07(3.53, 3.72)		2.57(2.09, 3.15)
*P*_trend_		< 0.001		< 0.001
Q1(< 1.5)	136(11.0)	1.00 (ref.)		1.00 (ref.)
Q2(1.5-3.0)	148(12.3)	1.13(0.88, 1.45)		1.07(0.83, 1.37)
Q3(3.0-5.1)	209(17.2)	1.67(1.33, 2.11)		1.47(1.16, 1.86)
Q4(≥ 5.1)	348(28.6)	3.23(2.60, 4.01)		2.63(2.09, 3.30)
*P*_trend_		< 0.001		< 0.001

T, tertile; Q, quartile

Adjusted model: Adjusted for maternal age, pre-pregnancy BMI, educational level, parity, and family history of diabetes

### The role of hs-CRP in the association between pregnancy loss history and GDM

The mediating effect of hs-CRP on the association between pregnancy loss history and risk of GDM in subsequent pregnancies is shown in Table 4. We observed that pregnancy loss history had an indirect effect on the GDM risk mediated by increased hs-CRP concentration. The total, direct, and indirect effects were 0.31 (95% CI: 0.07, 0.56), 0.23(95% CI: 0.03, 0.44), and 0.08(95% CI:0.04, 0.12), respectively. The proportion of indirect effect was 25.6%. In addition, an approximate 20.4% mediating effect mediated by hs-CRP was found in abortion history, but not yet in miscarriage.

**Table 4:** Mediation analysis of hs-CRP on the association of pregnancy loss history with GDM

Effects	Pregnancy loss	Induced abortion	Miscarriage
Total effect	0.31(0.07, 0.56)	0.27(0.12, 0.42)	0.07(-0.15, 0.28)
Direct effect	0.23(0.03, 0.44)	0.22(0.09, 0.34)	0.07(-0.12, 0.25)
Indirect effect	0.08(0.04, 0.12)	0.05(0.03, 0.08)	0.00(-0.03, 0.04)
Proportion mediated (%)	25.6	20.4	^

The model was adjusted for maternal age, pre-pregnancy BMI, educational level, parity, and family history of diabetes

^ Mediating effects not significant

## Discussion

In this prospective birth cohort study, we test the mediation role of hs-CRP in the biological pathways underlying the relationship between pregnancy loss history and GDM in a Chinese pregnant population. Our results found that abortion history was associated with a higher risk of GDM and an increase in the number of abortion histories. Additionally, the mediation analysis indicated this association was mediated by an increased hs-CRP level with a 20.4-25.6% of indirect effect ratio. However, there was no association between miscarriage and GDM. These findings further highlight how adverse reproductive events such as early chronic stress can cause a higher risk of GDM.

Miscarriage is the most common adverse outcome of early pregnancy. Some evidence suggests that miscarriage is associated with increased oxidative stress and inflammation, which may be the pathophysiological mechanism for the development of GDM [19, 20]. In addition, a recent cohort study in China reported that a history of miscarriage was associated with an increased risk of gestational diabetes in subsequent pregnancies [14]. However, in our study, we did not find these associations between the number of miscarriage and hs-CRP concentration and GDM risk. The main reason may be that the etiology of miscarriage is a multifactorial process, and some confounding factors including genetics, infection, and stressful life event might affect the association between miscarriage and GDM [21]. Moreover, due to our sample may not have enough statistical power.

Abortion is a worldwide public health issue and also worth remarking on pregnancy events. We are aware of one previous study that reported the associations of abortions with metabolic syndromes in middle-aged and elderly among 10 375 Chinese women from a cross-sectional survey [13]. In some South and North American countries, such as Brazil and Mexico, abortion is largely illegal, which may be cultural and religious beliefs [22, 23]. Thus, to date, studies on the association of abortion with GDM risk were rare in Europe and North America. However, our study addresses these evidence gaps by investigating the history of previous abortion in Chinese pregnant women. Our finding observe a dose-response effect, with elevated GDM risk associated with increased exposure to abortions. This provides good evidence for concluding that there is causal relationship. Obviously, additional studies should investigate this further, but given the limits of our study, this observed dose effect is a strong indication that our findings are not due to incidental effects.

In the present mediation analysis, we found that the association between abortion history and GDM risk was interpreted by the increased hs-CRP. Our findings indicated that hs-CRP may be a potential mechanism for the association of GDM about previous abortion history exposure. The inflammatory response regulates the maternal immune system through inflammatory factors, in pregnancy abortio [24]. Recurrent miscarriage is associated with disruption of the balance between pro- and anti-inflammatory T cells [25, 26]. Epidemiological evidence showed that women with a history of recurrent miscarriage had significantly higher levels of hs-CRP compared to those without miscarriage, which suggested a pro-inflammatory state in women with recurrent miscarriage history [27]. Given the similar pathology of GDM and T2DM, inflammation plays to establish a crucial role in the induction of insulin resistance, resulting in hyperglycemia and GDM development [28]. Evidence of inflammatory dysregulation can be found in pregnant women with GDM [29].

This study had several strengths.First, based on a prospective cohort design, we found an association between abortion history and GDM, expanding the research horizon of pregnancy loss history and the risk of GDM. Second, this was the first study to investigate the mediating role of hs-CRP in the association between abortion history and GDM risk, which would provide a potential biological pathway that links abortion history to GDM. Third, Given the adverse effects of GDM on the short- and long-term health of mothers and their offspring, the findings of this study may also have potential implications for public health.

Several limitations of this study need consideration. First, the data of abortion histories in medical records were based on self-reported and may have been subject to recall and reporting bias, resulting in misclassification of the exposure status. Therefore, our findings may be more likely to underestimate than to overestimate the effects associated with abortion. Second, there was a lack of data on post-abortion-related infections in this study. Considering that abortion-related infections may also contribute to subsequent GDM, future research, especially record-based studies, should be explored. Third, our data did not identify which abortions were surgical or medically induced. We were unable to shed light on any difference in the observed outcomes between surgical and medical abortion, which will be an important topic for future research. Fourth, although we adjusted for potential confounders, we could not rule out the potential residual effects from unmeasured or undefined confounders. Fifth, considering this is an observational study, the result should be interpreted with caution as we are unsure of the causal associations. Sixth, all participating pregnant women were from a city, which may limit the generalizability of our findings.

## Conclusions

This cohort study observed that a dose-response effect, with elevated GDM risk associated with increased exposure to abortions. In addition, hs-CRP had a mediation effect in the pathways linking abortion history with GDM. These findings emphasize that pregnant women with a history of abortion should be concerned about the risk of GDM, and early monitoring of blood glucose during pregnancy should be considered for prevention. Given the possible association of GDM with the risk of developing type 2 diabetes later in life, future studies should investigate whether abortion associated GDM is more or less associated with the subsequent development of type 2 diabetes.

## Data Availability

The datasets generated and/or analyzed during the current study are not publicly available but are available from the corresponding author on reasonable request.

## Abbreviations

GDM: Gestational diabetes mellitus
hs-CRP: High-sensitivity C-reactive protein
RR: Relative risk
CI: Confidence interval
OGTT: Oral-glucose-tolerance test
FPG: Fasting plasma glucose
1-h PG: 1 h plasma glucose
2-h PG: 2 h plasma glucose
